# Respiratory anomalies associated with gadoxetate disodium and gadoterate meglumine: compressed sensing MRI revealing physiologic phenomena during the entire injection cycle

**DOI:** 10.1007/s00330-021-08114-2

**Published:** 2021-07-29

**Authors:** Carl Guillaume Glessgen, Hanns-Christian Breit, Tobias Kai Block, Elmar Max Merkle, Tobias Heye, Daniel Tobias Boll

**Affiliations:** 1 Department of Radiology, University Hospital Basel, Basel, Switzerland; 2 Center for Advanced Imaging Innovation and Research, Department of Radiology, New York University Grossman School of Medicine, New York, USA

**Keywords:** Liver, Contrast media, Image processing, Respiratory rate

## Abstract

**Objectives:**

The goal of this study was to investigate the precise timeline of respiratory events occurring after the administration of two gadolinium-based contrast agents, gadoxetate disodium and gadoterate meglumine.

**Materials and methods:**

This retrospective study examined 497 patients subject to hepatobiliary imaging using the GRASP MRI technique (TR/TE = 4/2 ms; ST = 2.5 mm; 384 × 384 mm). Imaging was performed after administration of gadoxetate (*N* = 338) and gadoterate (*N* = 159). All GRASP datasets were reconstructed using a temporal resolution of 1 s. Four regions-of-interest (ROIs) were placed in the liver dome, the right and left cardiac ventricle, and abdominal aorta detecting liver displacement and increasing vascular signal intensities over time. Changes in hepatic intensity reflected respiratory dynamics in temporal correlation to the vascular contrast bolus.

**Results:**

In total, 216 (67%) and 41 (28%) patients presented with transient respiratory motion after administration of gadoxetate and gadoterate, respectively. The mean duration from start to acme of the respiratory episode was similar (*p* = 0.4) between gadoxetate (6.0 s) and gadoterate (5.6 s). Its mean onset in reference to contrast arrival in the right ventricle differed significantly (*p* < 0.001) between gadoxetate (15.3s) and gadoterate (1.8 s), analogously to peak inspiration timepoint in reference to the aortic enhancement arrival (gadoxetate: 0.9s *after*, gadoterate: 11.2 s *before* aortic enhancement, *p* < 0.001).

**Conclusions:**

The timepoint of occurrence of transient respiratory anomalies associated with gadoxetate disodium and gadoterate meglumine differs significantly between both contrast agents while the duration of the event remains similar.

**Key Points:**

• *Transient respiratory anomalies following the administration of gadoterate meglumine occurred during a time period usually not acquired in MR imaging.*

• *Transient respiratory anomalies following the administration of gadoxetate disodium occurred around the initiation of arterial phase imaging.*

• *The estimated duration of respiratory events was similar between both contrast agents.*

## Introduction

It has been established that administration of gadoxetate disodium may lead to transient episodes of respiratory inconsistencies, noticeable as breath-hold failures with subsequent tachypnea which possibly result in detrimental artifacts during arterial phase MR imaging [1, 2]. The incidence of this phenomenon associated with gadoxetate disodium administration has already been extensively assessed, specifically in comparison with other gadolinium-based contrast agents [3–10]. Additional factors, such as a dose-dependency of the phenomenon’s occurrence, have also been investigated [11, 12]. The respiratory anomalies appear to be limited to the arterial phase and to be more frequent with increasing contrast dose. However, the exact time point of manifestation within the injection cycle remains uncertain, as the required temporal resolution and coverage of the entire injection cycle have rarely been the focus of comparative MR investigations, as of yet.

Golden-angle radial sparse parallel (GRASP) MRI, which is a relatively new MRI technique based on the compressed-sensing principle, has shown the ability to assess respiratory and hemodynamic metrics related to different gadolinium-based contrast agents, even during free-breathing image acquisition [13]. As one specific feature of GRASP MRI, previously acquired raw datasets can be reformatted at various temporal resolutions, as low as 1 s of acquisition time. Also, the pre-injection and contrast arrival phases, both of which are usually not included in clinical reconstructions, can be retrieved analogously.

The aim of this study was to utilize this reconstruction feature to investigate the precise timeline of respiratory event occurrences following the administration of two gadolinium-based contrast agents, gadoxetate disodium and gadoterate meglumine, by analyzing the entire contrast-material injection cycle.

## Materials and methods

Our institutional review board (IRB) approved this study; the initial patient recruitment was performed prospectively with patients providing written consent, and this study represented an extended post hoc analysis of the initial study results [13].

### Study population

**Fig. 1:**
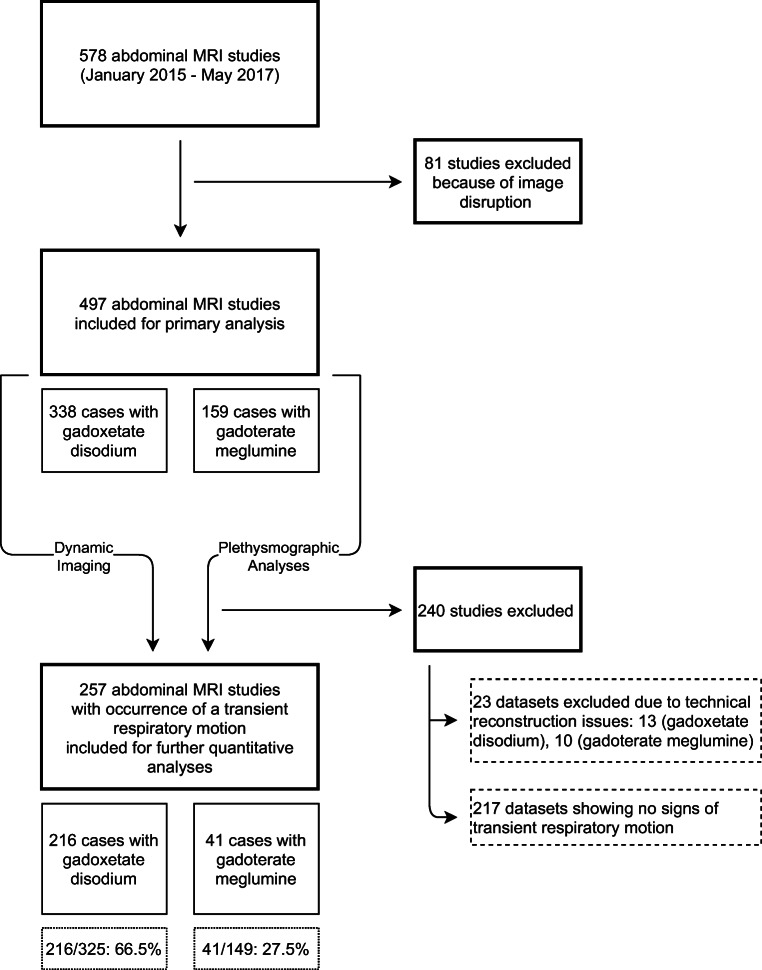
Flowchart outlining the selection of the final study group with the inclusion and exclusion criteria used within the defined observation window

Study participants were recruited from consecutively planned abdominal MRI studies performed during a 2.5-year timespan (01/2015–05/2017) at a single tertiary-care university hospital. Primary indication for MRI examination was either the investigation of focal liver lesions or the assessment of underlying cirrhosis. Exclusion criteria were incomplete raw data acquisition and physical patient motion unrelated to contrast administration during the examination. A total of 497 abdominal examinations in 434 different participants (207 women [mean age, 58 years; range, 14–87 years] and 227 men [mean age, 60 years; range, 19–91 years]) were included in the study, as shown in Fig. 1. Alternative reasons for potential respiratory irregularities, such as the presence of pleural effusions, ascites, or pulmonary disease, were noted.

### Contrast material

For MRI of focal liver lesions or cirrhosis, two different gadolinium-based contrast agents are used at our institution to evaluate dynamic enhancement in a free-breathing imaging protocol. In our study group, 338 participants received gadoxetate disodium (Primovist or Eovist; Bayer HealthCare Pharmaceuticals Inc.) and 159 participants received gadoterate meglumine (Dotarem; Guerbet).

Contrast material was administered weight-adjusted: for gadoxetate disodium, participants weighing less than 50 kg were administered 7.5 mL, participants weighing between 50–100 kg were administered 10 mL, and 15 mL was administered for participants weighing more than 100 kg; for gadoterate meglumine, a weight-adjusted concentration of 0.2 mL/kg was administered with an upper dose limit of 20 mL.

The contrast injection regimen for both contrast agents using a constant flow rate of 1 mL/s consisted of an initial 20-mL saline primer (NaCl 0.9, Braun Medical) with the injection starting with data acquisition, followed by the weight-adjusted volume of undiluted contrast material, and concluding with a 40-mL saline chaser, all injected peripheral-intravenously.

### MR parameters and image reconstruction

All examinations were performed using either 1.5-T or 3-T MR systems (MAGNETOM Avanto or MAGNETOM Skyra; Siemens Healthineers) employing DOT-engines to minimize the impact of operator variations. All participants were placed in a supine position with their arms alongside their bodies. Body phased-array coils with up to 48 channels were used for the examinations.

The dynamic contrast-enhanced acquisition used a fat-saturated T1-weighted radial stack-of-stars 3D GRE sequence with compressed-sensing and parallel-imaging reconstruction (GRASP) covering the entire liver with the following parameters: flip angle = 12°, repetition time ms/echo time = 4/2 ms, slice thickness = 2.5 mm, field of view = 384 × 384 mm, matrix = 288 × 288, in-plane resolution = 1.3 × 1.3 × 2.5 mm. The GRASP sequence was acquiring imaging raw data continuously for up to 270 s, starting with a 20-s non-contrast imaging phase prior to contrast-material administration and a subsequent contrast-enhanced imaging phase of up to 250 s. GRASP performs the continuous acquisition with a radial stack-of-stars k-space sampling scheme. This scheme enables retrospective reconstruction of image series with flexible temporal resolution by binning a certain number of consecutive spokes into individual time frames [14].

For this study, the image reconstruction used two sets of differing temporal k-space frames with each frame consisting of 5 spokes per frame for a 1^st^ segment with 450 spokes, and, subsequently, of 34 spokes per frame for a 2^nd^ segment until the end of the acquisition. The resulting temporal resolution of the reconstructed images from the dynamic scan was defined by the duration of each frame, which was 1 image per 1 s for the 1^st^ segment, using batches of five spokes each, and of 1 image per 7 s for the 2^nd^ segment, using batches of 34 spokes each. The 1^st^ segment covered the time window relevant for our analysis with a total duration of 80 s, while the 2^nd^ segment had various lengths, between 100 and 170 s, depending on the sequence’s final duration.

### Plethysmographic excursion derived from k-space data

**Fig. 2:**
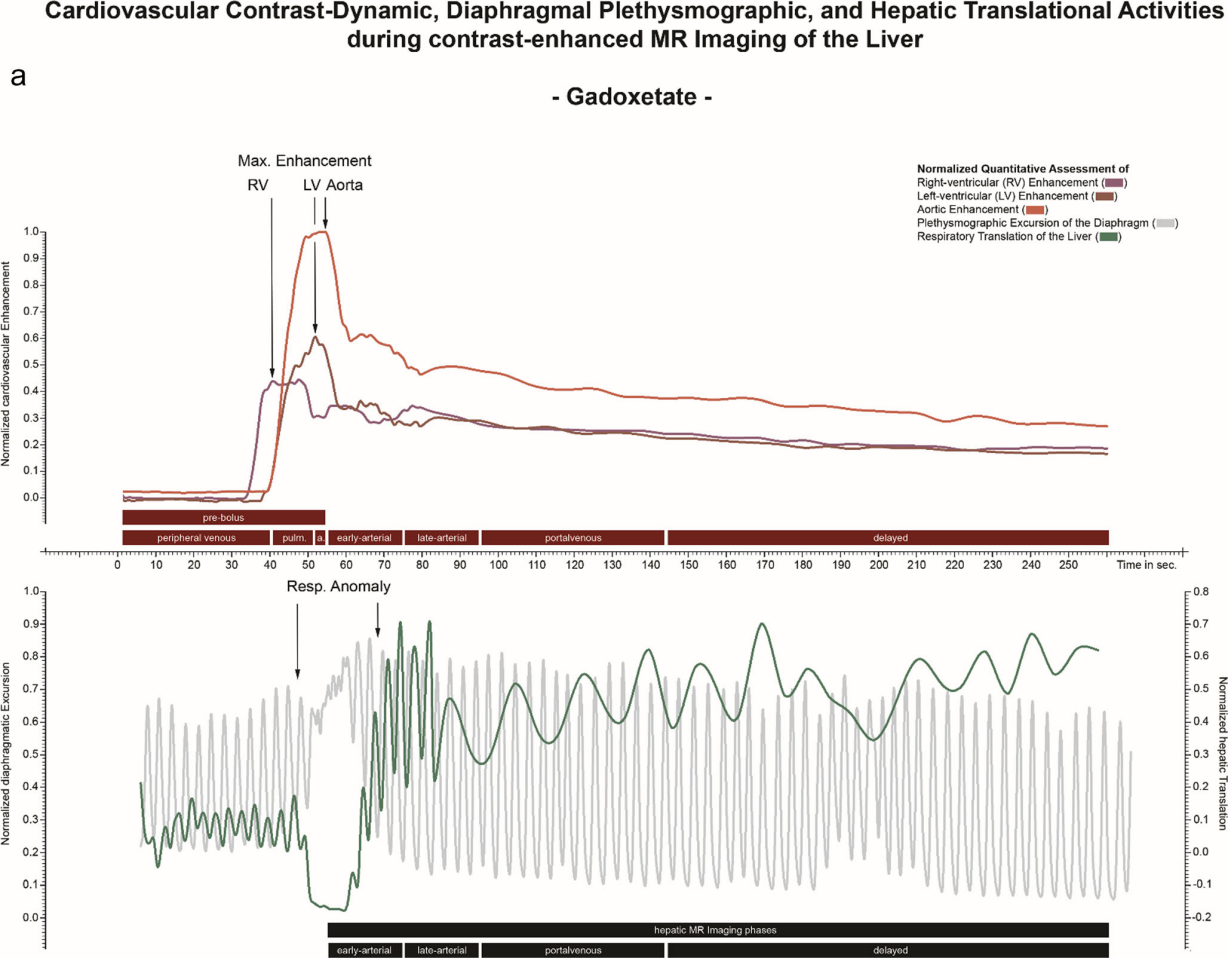

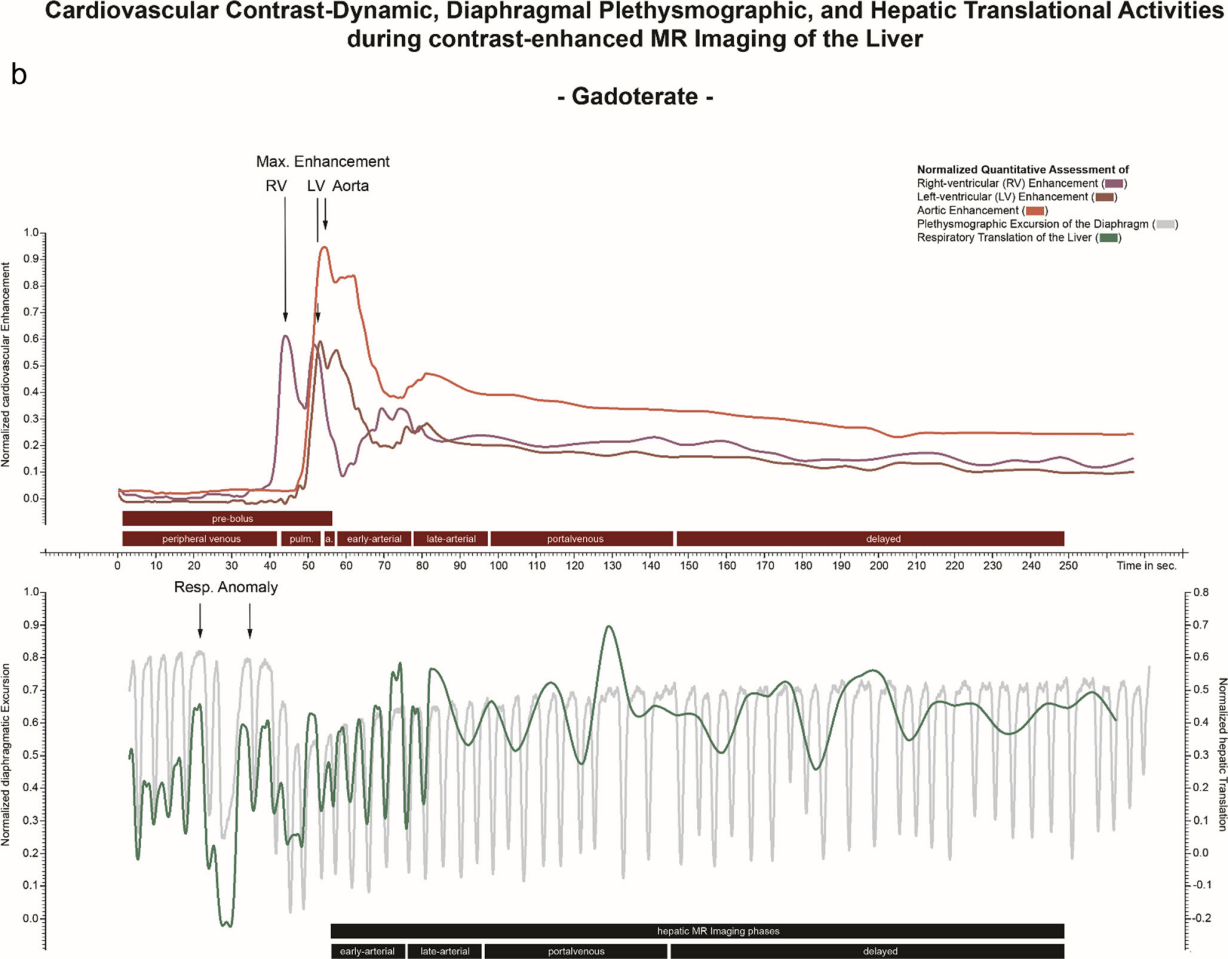
Superposed infographic map summarizing the cardiovascular contrast dynamic, the hepatic translational activities, and the diaphragmatic plethysmography during the contrast-enhanced MRI of the liver following administration of gadoxetate (**a**) and gadoterate (**b**), in two different patients. The relative cardiovascular intensity values normalized based on the initial 20-s acquisition extracted from right-ventricular (dark red), left-ventricular (red), and aortic (light red) ROIs plotted over time allow individual definition of eight time points throughout the election cycle (upper graph); temporal evolution is 1 s. The relative hepatic intensity values were derived from the hepatic ROI (green), plotted over time; temporal resolution is 1 s. A hepatic displacement implying a transient respiratory motion beyond normal breathing motion was considered to be significant when a variation of the signal intensity of at least two standard deviations from the baseline was observed. The extracted plethysmographic curve (gray) with a temporal resolution of 0.19 s is superimposed onto the hepatic intensity curve (lower graph). Pronounced respiratory irregularity is seen during the early arterial contrast phase for gadoxetate (**a**) and during the peripheral venous phase for gadoterate (**b**), both resulting in extensive hepatic translational movement. Transient respiratory motion occurring after administration of gadoterate (**b**) is outside the acquisition window for arterial phase MR imaging (black box) while gadoxetate (**a**) administration leads to motion during the crucial early arterial MR imaging phase

The computation of the plethysmographic maps from the GRASP sequence was performed with a soft-gating algorithm implemented in MATLAB (DICOM and Statistics Toolbox Release 2017b, The MathWorks). While the normal image reconstruction relies on multiple spokes combined into a single imaging frame per time point, k-space evaluation for diaphragmatic motion detection evaluated every change of k-space through the addition of individual new spokes throughout the entire imaging interval, resulting in a temporal resolution of 0.19 s for each updated map. Plethysmographic maps were combined into normalized respirational curves. The k-space evaluation technique and normalization procedure were performed analogously to the description in [13] (Fig. 2).

The resulting normalized plethysmographic curves were employed to detect respiratory motion following the administration of contrast material. Significant motion was defined as a variation of the duration from the maximum-to-maximum inspiration of at least two standard deviations from baseline (2 × 0.6482 s), over an interval of at least two breathing cycles. Examinations without detectable variation in respiration during these initial imaging phases were not further analyzed in this study (Fig. 1, Table 1).

**Table 1:** Demographic characteristics and main clinical confounders for both study arms. Values are presented as mean ± standard deviation unless indicated otherwise

Demographics and confounders	Gadoxetate *N* = 216	Gadoterate *N* = 41	*p* value
Age in years	60 ± 14.6	58 ± 16.6	0.5
Gender			
Female	111 (51%)	20 (49%)	0.9
Male	105 (49%)	21 (51%)	
Weight in kg	77.1 ± 17.5	72.1 ± 16.8	0.1
Body mass index in kg/m^2^	26.9 ± 5.7	25.2 ± 4.9	0.05
Presence of cirrhosis	52 (24%)	3 (7%)	0.001
Presence of effusions	8 (3%)	1 (2%)	0.7
Presence of ascites	31 (14%)	2 (5%)	0.02
Combined presence of ascites and effusions	5 (2%)	2 (4%)	0.5
History of pulmonary disease			
Chronic obstructive pulmonary disease	2 (1%)	1 (2%)	0.6
Lung cancer	6 (3%)	0	0.01
Asthma	0	1 (2%)	0.3

### Quantitative assessment

Image datasets with detectable breathing changes were quantitatively analyzed in a dual approach: (a) evaluations of the cardiovascular contrast dynamics and (b) of respiratory-induced translation of the liver. The quantitative assessments were performed using a commercially available postprocessing software application (syngo.via VB30; Siemens Healthineers). Evaluations were performed in consensus readings by two radiologists, C.G.G. and H.-C.B., both with 3 years of experience in image postprocessing, on de-identified image datasets and without annotations regarding the administered contrast agent.

#### Evaluation of cardiovascular contrast dynamics

Each four-dimensional dataset was loaded into the software application and reformatted into the axial and coronal image planes. Three regions-of-interest (ROIs) were placed manually: within the central right and left ventricles, and within the lumen of the abdominal aorta immediately upstream from the origin of the celiac trunk. The resulting intensity values were normalized and plotted over time as presented in Fig. 2. These cardiovascular intensity curves resulted in eight intra-individually defined contrast phases: T_baseline_ (first 20 s of the acquisition), T_peripheral-venous_ (peripheral-venous distribution starting at the 20-s mark until right-ventricular maximum intensity), T_pulmonary_ (pulmonary distribution ranging from right-ventricular to left-ventricular intensity maxima), T_aortic_ (aortic distribution ranging from left-ventricular to aortic maxima), T_early-arterial_ and T_late-arterial_ (hepatic arterial parenchymal distribution during two consecutive 20-s intervals starting at maximum aortic intensity), T_portal venous_ (portal venous parenchymal distribution from 40 to 90 s after maximum aortic intensity), and finally T_delayed_ (contrast distribution from 90 s after maximum aortic intensity up to the end of the acquisition). The first four contrast phases will be referred to as the pre-bolus phase.

In addition, relative cardiovascular curves were employed to assess cardiovascular function through time intervals, specifically (1) right-ventricular to aortic peak enhancement (surrogate for global cardiac function), (2) right-ventricular to left-ventricular peak enhancement (surrogate for right cardiac function), and (3) left-ventricular to aortic peak enhancement (surrogate for left cardiac function).

#### Evaluation of respiratory-induced translation of the liver

Each four-dimensional image set was reformatted to the coronal plane for evaluation of hepatic cranio-caudal translation due to respiration. A crescent-shaped polygonal ROI was placed just below the diaphragm within hepatic segment VIII during expiration-induced buckling of the right diaphragm. The intensity values were normalized and plotted over time. Hepatic translation was represented by decreasing signal intensities as seen in Fig. 2.

Hepatic displacement implying a transient respiratory motion was quantified with respect to (1) its duration from onset to peak inspiration—acme—(2), its onset timepoint, and (3) its acme timepoint. Values for (2) and (3) were measured in reference to the right-ventricular contrast arrival and the peak aortic enhancement, respectively. The onset of the hepatic displacement was determined by a negative downslope of the intensity curve over at least five measured time points below the baseline.

### Statistical analysis

All quantitative results from the evaluation of the cardiovascular contrast dynamics and respiratory translation of the liver are presented as mean ± standard deviation (SD). The analysis of variance (ANOVA) methodology was used for statistical comparison. The type of contrast material was defined as an independent factor for each analysis. Bonferroni post hoc corrections were applied for the fixed factor “contrast phase.”

For the ANOVA, the following parameters were successively selected as dependent variables: mean duration of each vascular cycle during the pre-bolus phase (s), mean duration of hepatic translation (s), timepoint of onset of hepatic translation relative to right-ventricular contrast arrival (s), and timepoint of maximal hepatic translation relative to right-ventricular contrast arrival (s).

Statistical analyses were performed using SPSS (SPSS Statistics 21, SPSS Inc.). A *p* value < 0.05 was considered statistically significant, and the resulting Bonferroni threshold was 0.05/4 = 0.0125.

## Results

### Study population

The final study population included for analyses comprised 257 patients of which 216 received gadoxetate disodium and 41 received gadoterate meglumine (Fig. 1). There was neither a significant difference in gender distribution between patients who received gadoxetate disodium and gadoterate meglumine (51 % and 49 % female patients, respectively, *p* = 0.9), nor was there a significant difference in age (60 years and 58 years, respectively, *p* = 0.5), mean weight (77.1 kg and 72.1 kg, respectively, *p* = 0.1), or body mass index (BMI of 26.9 and 25.2 kg/m^2^, respectively, *p* = 0.05) (Table 1). The participants who received gadoxetate disodium had a higher incidence of global liver diseases such as cirrhosis than those who received gadoterate meglumine (*p* = 0.001). Effusions, ascites, and pulmonary disease were similar in distribution between both contrast groups (Table 1).

### Cardiovascular contrast dynamics

The duration of the actual intravenous contrast injection was shorter for gadoxetate disodium with 10.0 s compared to 13.4 s for gadoterate, *p <* 0.001. The time period from right-ventricular contrast peak to aortic enhancement peak was 10.5 s for gadoxetate disodium and 10.4 s for gadoterate meglumine, *p* > 0.9. The duration of pulmonary transit was 7.5 s versus 7.3 s for gadoxetate disodium and gadoterate meglumine, respectively, *p* = 0.6. The time period of aortic transit of the contrast agent as a surrogate for left-ventricular function was 3.2 s versus 2.9 s for gadoxetate disodium and gadoterate meglumine, respectively, *p* = 0.6 (Table 2).

**Table 2:** Overview of the estimated cardiac output for both study arms. Values are presented as mean ± standard deviation. *RV*, right ventricle; *LV*, left ventricle; *Ao*, abdominal aorta

Cardiovascular contrast dynamics		Gadoxetate *N* = 216	Gadoterate *N* = 41	*p* value
Contrast injection duration, in seconds		10.0 ± 1.1	13.4 ± 3.6	< 0.001
Surrogate for *global* cardiac function (enhancement time from RV to Ao), in seconds		10.5 ± 3.6	10.4 ± 3.0	> 0.9
• In patients with cirrhosis 10.2 ± 3.3 • In patients without cirrhosis 10.3 ± 3.9	*p* = 0.8			
Surrogate for *right* ventricular function, or pulmonary transition time (enhancement time from RV to LV), in seconds		7.5 ± 3.0	7.3 ± 2.5	0.6
Surrogate for *left* ventricular function (enhancement time from LV to Ao), in seconds		3.2 ± 2.5	2.9 ± 2.1	0.6

### Respiratory-induced translation of the liver

From the initial group of 497 patients evaluated for the occurrence of a disruption in the plethysmograph navigator, 257 patients presented with a transient respiratory anomaly of which 216 received gadoxetate disodium (216/325, 66.5%) and 41 received gadoterate meglumine (41/149, 27.5%).

The duration of hepatic translation, from onset to acme, was similar between both groups, 6.0 s for gadoxetate disodium and 5.6 s for gadoterate meglumine, *p =* 0.4.

The mean onset of the event was 15.3 s *after* right-ventricular contrast arrival, for gadoxetate disodium, and 1.8 s *after* right-ventricular contrast arrival for gadoterate meglumine, *p* < 0.001.

The acme of the transient respiratory anomaly recognizable as the deepest respiratory-induced excursion of the diaphragm occurred on average 0.9 s *after* peak aortic enhancement for gadoxetate disodium, and 11.2 s *before* peak aortic enhancement for gadoterate meglumine, *p* < 0.001 (Table 3).

**Table 3:** Main characteristics of the observed transient liver motion for both study arms. Values are presented as mean ± standard deviation

Respiratory-induced translation of the liver	Gadoxetate *N* = 216	Gadoterate *N* = 41	*p* value
*Duration* of transient hepatic translation	6.0 ± 2.2	5.6 ± 2.7	0.4
*Onset* of hepatic translation in reference to contrast arrival in the right ventricle	15.3 ± 4.5	1.8 ± 9.8	< 0.001
*Acme* of hepatic motion in reference to aortic enhancement peak	0.9 ± 4.1	−11.2 ± 10.8	< 0.001

## Discussion

This study investigated the precise timeline of respiratory events following the administration of two gadolinium-based contrast agents, gadoxetate disodium and gadoterate meglumine. The high temporal resolution and large temporal coverage we utilized brought to light interesting insights regarding transient episodes of respiratory anomalies following contrast administration. In particular, the pre-bolus and contrast-arrival phases have been evaluated, which are usually not included in clinical acquisitions.

The onset of respiratory anomalies was markedly affected by the underlying type of contrast material administered. Patients in the gadoxetate group presented with an onset of respiratory anomalies ranging from 11 to 19 s after right-ventricular contrast arrival, significantly later compared to patients in the gadoterate group, with an onset ranging from 8 s before to 10 s after right-ventricular contrast arrival. This suggests differing trigger locations and/or mechanisms for this phenomenon, in particular when considering further delays between initial trigger and subsequent detected effects. For example, transient severe motion occurring with gadoterate before it reaches the aorta implies that it is not triggered by the brain or any arterial chemoreceptors in the upstream aorta. Conversely, pulmonary or venous trigger locations could be the cause. Yet, the impact and precise mechanism of peripheral venous chemoreceptors is still a field of ongoing investigation [15]. Of importance, however, is the fact that not a single transient respiratory motion event was detected prior to the presence of gadolinium-containing contrast material in the vascular system.

The deepest respiratory-induced excursion of the diaphragm resulting in the most extreme translation of the liver occurring during the first-pass contrast cycle most likely represents the somatic equivalent of the visualized artifacts during arterial phase imaging. The acme of hepatic motion in reference to the aortic enhancement peak occurred at significantly different time points. While for patients receiving gadoterate, this event occurred on average 11 s *before* initiation of arterial phase imaging, thereby granting patients time to normalize their breathing pattern thereafter, patients receiving gadoxetate experienced the transient respiratory anomaly concurrently *with* the initiation time of arterial phase imaging. Therefore, as of yet, transient respiratory anomalies appear to occur almost exclusively following administration of gadoxetate in contrast to any other gadolinium-containing contrast material as described in previous studies [1, 4, 6–9].

The duration from onset to maximum of the transient respiratory motion did not differ significantly between both groups and was found to be 6.0 s for gadoxetate disodium and 5.6 s for gadoterate meglumine. Previous studies’ approximations of the whole duration (onset to normalization) of transient hepatic motion ranged between 17 and 21 s [5, 13]. To determine the exact complete duration, observers face the challenge to determine the onset as well as the return to baseline. While the former may be detected in a reproducible fashion using quantitative assessment, the latter is more challenging to determine as no abrupt return to baseline was observed in our approach. To achieve reproducibility, we used the duration between onset to acme of hepatic motion in our assessment and hereby utilized clearly identifiable time points. This choice, however, explains the discrepancy between durations of transient respiratory anomalies in our study compared to previous studies’ results.

Regarding the rate of detected transient respiratory motion, we have found higher incidences than previously reported, i.e., in 67% of patients who received gadoxetate and in 28% of patients who were administrated gadoterate meglumine. While a related quantitative study reported incidences of 71% and 29% for gadoxetate and gadoterate, the vast majority of earlier works reported frequencies ranging from 2 to 40% and from 0 to 10% for gadoxetate and gadoterate, respectively [1, 2, 4–9, 16–18]. Potential reasons for this discrepancy are certainly related to the more sensitive quantitative method applied in this study, which also detects respiratory anomalies that not necessarily lead to imaging artifacts during the arterial phase but that are still noticeable on plethysmographic as well as hepatic translational analyses [6].

It has to be noted that we addressed confounding factors such as possible differences in cardiovascular function in order to validly discuss the exact timeline of effects potentially related to first-pass pharmacodynamics of the contrast agents. Our study employed the clearly visible contrast bolus at defined and reproducible anatomic locations as a surrogate to evaluate right-ventricular, left-ventricular, and global cardiac function. This scheme hereby builds on the methodology applied by previous MR angiography assessments of cardiopulmonary transit times [19, 20]. The injection duration was slightly longer for gadoterate due to overall larger administered volumes, but nonetheless, respiratory anomalies under gadoterate occurred earlier. Consequently, we do not expect a relevant bias to be present originating from actual injection time durations. Also, there was no difference in our surrogate for global cardiac function between patients. As no further differences were detected amongst the cardiovascular dynamics, we assume that existing interindividual differences in cardiovascular function are of nonsignificant importance.

Our study has limitations that need to be acknowledged. First, the assessment of respiratory motion from dynamic imaging scans with high time resolution has not been fully validated against non-imaging-based methods such as standardized plethysmography. Second, our approach uses surrogates for evaluating the respiratory as well as cardiovascular function. Each surrogate is based on assumptions, which have not been confirmed by non-imaging-based standardized methodologies. Third, our study evaluates only two different gadolinium-based contrast agents, which implies limited generalization. However, these two types, structured linearly with intracellular uptake for gadoxetate disodium and macrocyclic with mostly extracellular distribution for gadoterate meglumine, are the most used at our institution and mirror the daily practice of a quaternary care hospital system. We assume other institutions to have similar practice schemes. Finally, each patient did not present as his or her own control having received both contrast agents.

In summary, this study showed that administration of various types of gadolinium-based contrast agents leads to respiratory irregularities under free-breathing conditions, which mainly differ in their timepoint of occurrence. No difference was detected in the duration from their onset to maximum, between the two evaluated gadolinium-based contrast materials. We expect future research to focus on ways to bypass the peak of the respiratory anomalies, as Pyetriga et al successfully did by acquiring three arterial phases instead of one [16]. While respiratory anomalies which critically impair images remain intermittent, researching how to dispose of them could prove useful in other settings where artifacts occur at such a precise and predictable moment.

## Abbreviations

GBCA: Gadolinium-based contrast agent
GRASP: Golden-angle radial sparse parallel
